# Investigation on potential malaria vectors (*Anopheles* spp.) in the Province of Trento, Italy

**DOI:** 10.1186/s12936-019-2785-z

**Published:** 2019-04-29

**Authors:** Valentina Tagliapietra, Daniele Arnoldi, Marco Di Luca, Luciano Toma, Annapaola Rizzoli

**Affiliations:** 1 Fondazione Edmund Mach, Research and Innovation Centre, Via E. Mach 1, San Michele all’Adige, Trento Italy; 2 Department of Infectious Diseases, Istituto Superiore di Sanità, Viale Regina Elena, Rome, Italy

**Keywords:** Malaria vectors, *Anopheles* spp., Province of Trento, Italy

## Abstract

**Background:**

Europe and Italy were declared malaria free since the 1970s although the presence of competent vectors and the high number of yearly imported malaria cases make this disease a potential rising health issue. In September 2017, a cryptic fatal case of *Plasmodium falciparum* malaria in the Province of Trento, Italy, raised the concern of health authorities on the possible resurgence of this disease in the Mediterranean Basin.

**Methods:**

An entomological surveillance by means of BG traps, CDC light traps and larval search was performed. Sites were chosen among urban and suburban environments (e.g. private houses, public parks, schools, cemeteries, ecotone urban/forest, farms), ranging from an altitude of 91 to 1332 m above sea level. All the mosquitoes collected were morphologically identified and about half of them (103; 49%) were confirmed with the sequencing analysis of the rRNA internal transcribed spacer 2 (ITS-2).

**Results:**

In the present study 287 sites were screened for the presence of *Anopheles* spp. and 211 specimens were collected and identified. Hundred-eighteen individuals (56%) belonged to *Anopheles plumbeus*, 56 (26.5%) to *Anopheles maculipennis* complex, 10 (4.7%) to *Anopheles claviger* and 27 were identified only at genus level. This is the first record for the presence of *An. plumbeus* in the study area.

**Conclusions:**

The presence of Anopheles spp. mosquitoes in the Province of Trento, Italy, has been updated with the occurrence of *An. plumbeus*. The risk of malaria endemicity in the area is to be considered very low, but urban and peri-urban habitat may act as potential breeding sites for the presence of mosquito vectors and should be constantly monitored.

## Background

Europe and Italy were declared malaria free since the 70 s although the presence of competent vectors, mainly belonging to the *Anopheles maculipennis* complex, and the high number of yearly imported malaria cases make this disease a potential rising health issue [1]. In September 2017, an autochthonous fatal case of *Plasmodium falciparum* malaria in the Province of Trento, Italy—later confirmed as due to hospital-acquired infection [2]—attracted the attention of the media, health authorities and scientific community about the potential occurrence of malaria vectors in this area and in the whole Mediterranean Basin.

Several *Anopheles* species (Diptera, Culicidae) with variable susceptibility and capability to transmit the infection by *Plasmodium* spp. occur widely all over Europe, even if their exact distribution is still poorly known. The most important belong to the *An. maculipennis* complex (*An. maculipennis* sensu lato), while others (*Anopheles algeriensis*, *Anopheles claviger*, *Anopheles hyrcanus*, *Anopheles plumbeus*, *Anopheles superpictus*) have historically played a minor role as secondary vectors, although their vectorial competence is being reevaluated [1]. In particular, *An. plumbeus* proved to have some receptivity towards *P. falciparum* and, therefore, it was suspected to be involved in cryptic malaria transmission in Central-Western Europe [3].

In Italy, the presence of different *Anopheles* species is well-known only in very limited areas [4]. Conversely, in many regions the distribution of such species is completely unknown or should be updated, as targeted entomological surveys have been conducted only in the past [5]. In the present study, the results of a 10-year monitoring on the occurrence and distribution of *Anopheles* spp. mosquitoes in the Province of Trento, Italy are presented.

## Methods

**Fig. 1:**
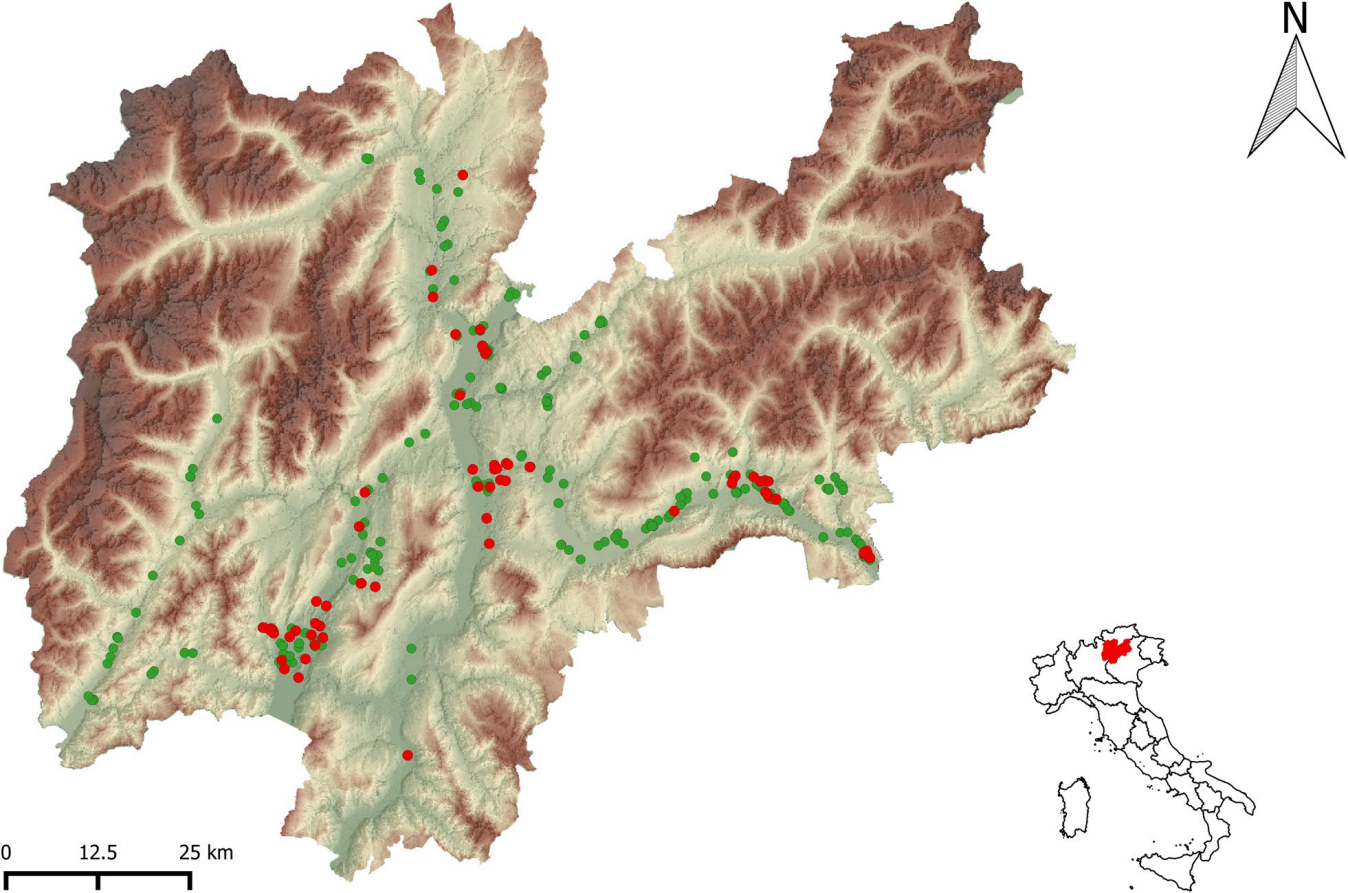
Distribution map of *Anopheles* spp in the Province of Trento (Green circles = negative sites for *Anopheles* spp.; Red circles = positive sites for *Anopheles* spp.)

An entomological surveillance on endemic and alien species of mosquitoes has been carried out from 2008 to 2018 in the Province of Trento, Italy, by means of BG traps (Biogents AG, Germany), CDC light traps (http://www.pepinfest.com) and larval search. The study area has an extension of 6212 km^2^, is mountainous, with more than 70% of the territory over 1000 m above sea level (a.s.l.), is forested, with 55% of the surface covered by coniferous and deciduous woods, and has a temperate-oceanic climate with four main areas: sub-Mediterranean (close to Lake Garda with mild winters), subcontinental (the main valleys with more severe winters), continental (the alpine valleys) and alpine (the areas above the tree line) [6]. A total of 287 sites were screened for the presence of immature or adult stages of *Anopheles* spp. (see Fig. 1). Trapping period started from the end of April to the beginning of November. BG and CDC light traps were always provided with lure (BG-Lure, Biogents AG, Germany), while CO_**2**_ (dry ice) was not regularly added to BG traps, according to the different projects running. Traps were activated during no windy or rainy days, and operated for 24 h with some exceptions (during years 2008 and 2009 in Arco and Riva del Garda traps were active 24/7 the whole season). In some sites (see Table 1), traps were run every other week for the whole trapping season, as part of a regular entomological surveillance, while in others they operated only once in the season (see Table 1). Larval search was performed in the period June–August. Sites were chosen among urban and suburban environments (e.g. private houses, public parks, schools, cemeteries, ecotone urban/forest, farms), ranging from an altitude of 91 to 1332 m a.s.l. All the individuals collected were morphologically identified [7, 8]. To confirm the reliability of species identification and assess a possible intraspecific heterogeneity, a random sample of 103 *Anopheles* specimens (49%) was processed, yielding ITS-2 sequences according to a previously described procedure [4, 9]. For 5 specimens of *An. plumbeus*, a fragment of the mtDNA COI (735 bp) was also characterized. COI and ITS-2 sequences were analyzed by DS gene 1.5 (Accelrys Inc., Cambridge), and then submitted to GenBank (accession no. MK618768-72; MK625341-6; MK625347-426; MK625428-33).

**Table 1:** *Anopheles* spp. collected in the Province of Trento (Italy) by species, sex and municipality (2008–2018)

Municipality/species	*An. claviger*	*An. maculipennis* s.l.		*An. maculipennis* s.s.		*An. plumbeus*		*Anopheles* spp.	
	F	F	M	F	M	F	M	F	M
ALA					1				
ARCO	3	27	5	1		6		12	4
CAMPODENNO^b^	1								
CASTEL IVANO^a^	1					40	1		
CAVEDINE^b^						1			
CIVEZZANO^a,b^									
COREDO^a,b^									
DENNO^b^	1								
DRENA^a,b^						1			
DRO						1		1	
GRIGNO	4			1		13		1	
MADRUZZO			1			1		1	
MEZZOCORONA						1			
MEZZOLOMBARDO^b^						1			
RIVA DEL GARDA		11	4			4		6	2
RONCEGNO TERME^a,b^									
SAN MICHELE A/A				2		6			
TELVE						8			
TENNO			1			16			
TRENTO		1		1		15	2		
ZAMBANA						1			
Total	10	39	11	5	1	115	3	21	6

s.l., sensu lato; s.s., sensu stricto; spp., *species pluralis*

^a^Presence of larval stage; F = adult female; M = adult male; An = Anopheles

^b^Sites visited only once in the study period 2008–2018

## Results

A total of 67 sites (23%; 67/287) resulted positive for the presence of immature or adult stages of *Anopheles* spp. (Fig. 1). In total 211 individuals were collected: 118 (56%) belonged to *An. plumbeus*, 56 (26.5%) to *An. maculipennis* complex, 10 (4.7%) to *An. claviger* and 27 were identified only at genus level (Table 1). Of note, the capture of *Anopheles* mosquitoes from our entomological surveillance, represents the 0.37% of all the mosquito samples collected. This is the first record for *An. plumbeus* in the study area.

About 49% (n = 103) of the samples already identified morphologically was processed at molecular level, generating ITS-2 sequences of *An. plumbeus* (n = 80), *An. maculipennis* s.s. (n = 6) and *An. claviger* (n = 6); unfortunately 11 sequences failed. For *An. plumbeus* and *An. maculipennis* sensu stricto (s.s.) no intraspecific variations were detected in the samples examined, whereas three variable sites were detected for *An. claviger*, identifying three haplotypes. In addition, the five COI sequences of *An. plumbeus* shared 100% identity with a sequence of such species from Austria (KM280577).

## Discussion

At the end of the 19th century Italy was one of the most endemic European countries for malaria with about 15,000–20,000 deaths per year [10]. As a result of an extensive and integrated approach to fight against this disease, Italy was declared malaria-free by the World Health Organization in 1970. Since then, the Italian surveillance system detected a rise in the malaria cases (700 per year from 2000 to 2008 [11]). Nonetheless, the malariogenic potential in Italy has to be considered low [12] due to the very small number of authochtonous cases (0.1% from 2000 to 2008 [11]), and the few and scattered mosquito species that can transmit *Plasmodium* spp. The lack of detailed or updated data in some areas of Italy makes it crucial to fill the gaps and improve the surveillance system in order to better assess possible risks.

The Province of Trento borders with historically known and suitable malaria areas such as the Padana valley, but has never been considered important for mosquito presence because of its mountainous features. Due to this, the only previous data on *Anopheles* spp. distribution, dates back to the 60 s [13] and reported the presence of *An. maculipennis* s.s. and *An. claviger*, both species considered refractory to *Plasmodium* spp. transmission [3, 26]. In this study it has been confirmed the presence of the above-mentioned species, but the presence of *An. plumbeus* has been recorded for the first time.

*Anopheles plumbeus* is a Palearctic species widely distributed throughout Europe (except the far northern regions), the Middle East and North Africa [7]. It can be found in forested areas from sea level up to 1200 m a.s.l. [14], where it preferentially breeds in water-filled tree holes, although females can also exploit other artificial breeding sites where to lay the eggs such as catch basins, tires, cemetery vases and domestic and peridomestic containers near human settlements, especially large manure pits [15]. Among all the specimens collected in this survey, *An. plumbeus* was the most abundant and wide distributed, although at low density. This species has recently become important for its role in malaria cryptic transmission cycles, provided the presence of gametocyte carriers in the area [3]. In particular its susceptibility to *P. falciparum* in experimental infections [3, 16, 17] and its longevity [3] that enables the *Plasmodium* parasites to complete their development to the oocyst and sporozoite stages make it suitable as a potential malaria vector [18]. Moreover, the ability to exploit man-made artificial breeding habitat and its anthropophilic behaviour, may favour an increase of the population numbers and its nuisance, as recently reported in Belgium [15], with potential consequences on public health risk. Furthermore, *An. plumbeus* can also produce sporozoites of *Plasmodium vivax* [19, 20] and some studies reported that it may have a considerable role in the maintainance of West Nile virus or even dirofilariasis [7, 21].

*Anopheles maculipennis* s.s. is the only species belonging to the *An. maculipennis* complex previously reported in the Province of Trento [13]. Among the collected individuals, 11% were molecularly analysed and confirmed as *An. maculipennis* s.s. Although this group has been historically recognized important for its involvement in the malaria transmission in Europe, it was shown that afrotropical *P. falciparum* strains—cause of the vast majority of imported malaria in Europe—are not well adapted to this complex of species [22–24]. Finally, *An. claviger* which is found throughout the Palearctic and is quite widespread in Italy [13, 25], is considered only an occasional vector for malaria [26, 27]. An evident intraspecific polymorphism has been found in *An. claviger,* which needs to be further investigated. The presence of individuals belonging to *An. maculipennis* s.s. and *An. claviger* in the Province of Trento (Italy) is to be considered negligible.

*Plasmodium* spp. transmission in malaria-free countries by local *Anopheles* mosquito species has progressively increased due to global changes and mass population movements [28]. In particular, *P. falciparum* completion of the sporogonic cycle in a gametocyte-fed, wild *An. plumbeus* requires an extended and continuous period of elevated temperatures. The recorded global increase in frequency, intensity and duration of heat events could affect it, however the epidemiological settings for malaria are not only linked to climatic factors. Following the fatal case reported in September 2017 and the lack of updated information on the distribution of *Anopheles* spp. in the Province of Trento (Italy) [13], this study represents a first step toward a better knowledge on the presence of this genus. Moreover, recent outbreaks of other mosquito-borne diseases other than malaria (e.g. West Nile, Chikungunya and Dengue viruses), demonstrate the importance of gathering as much information as possible on potential vectors presence and distribution, and this holds true especially for invasive mosquito species than can have both an economic and a human health impact.

## Conclusions

The aim of this study was to investigate the potential spatial distribution of Anopheles spp. mosquitoes in the Province of Trento, Italy. The presence of *An. plumbeus*, a potential malaria vector, has been recorded, but by now, the rare presence of the vector, the good malaria notification system, the prompt treatment of patients with imported malaria and the low probability of the vector to encounter a gametocyte carrier, are unlikely to facilitate an epidemic in the area. Nonetheless, as the global situation is dynamic and variable, gardens and urban parks, as well as other potential breeding sites such as catch basins or septic tanks, should be constantly monitored for the presence of mosquitoes, to provide updated information on the occurrence and dynamic of malaria and other emerging mosquito-borne diseases vectors.

## Abbreviations

An: Anopheles
s.l.: sensu lato
s.s.: sensu stricto
spp.: species pluralis
P: Plasmodium
